# High Precision Temperature Insensitive Strain Sensor Based on Fiber-Optic Delay

**DOI:** 10.3390/s17051005

**Published:** 2017-05-02

**Authors:** Ning Yang, Jun Su, Zhiqiang Fan, Qi Qiu

**Affiliations:** School of OptoElectronic information, University of Electronic Science and Technology of China, Chengdu 610054, China; ningchenluck@126.com (N.Y.); jianfzq@gmail.com (Z.F.); qqiu@uestc.edu.cn (Q.Q.)

**Keywords:** fiber-optic delay, strain sensor, temperature insensitive, elastic coefficient

## Abstract

A fiber-optic delay based strain sensor with high precision and temperature insensitivity was reported, which works on detecting the delay induced by strain instead of spectrum. In order to analyze the working principle of this sensor, the elastic property of fiber-optic delay was theoretically researched and the elastic coefficient was measured as 3.78 ps/km·με. In this sensor, an extra reference path was introduced to simplify the measurement of delay and resist the cross-effect of environmental temperature. Utilizing an optical fiber stretcher driven by piezoelectric ceramics, the performance of this strain sensor was tested. The experimental results demonstrate that temperature fluctuations contribute little to the strain error and that the calculated strain sensitivity is as high as 4.75 με in the range of 350 με. As a result, this strain sensor is proved to be feasible and practical, which is appropriate for strain measurement in a simple and economical way. Furthermore, on basis of this sensor, the quasi-distributed measurement could be also easily realized by wavelength division multiplexing and wavelength addressing for long-distance structure health and security monitoring.

## 1. Introduction

In order to ensure the safety of personal and public property, the precise and real-time monitoring of strain becomes more and more important in all kinds of engineering applications, such as chemical plants, gas stations, power stations, bridges, tunnels, oil pipelines, etc. [1,2,3,4,5]. In general, these application environments full of poisonous gas, intense radiation, and elevated temperature are dangerous to human health, so safe and efficient remote monitoring of strain is of great significance. Compared with conventional electrical sensing methods, an optical fiber strain sensor is more suitable for present applications because of its compact size, high sensitivity, multiplexing capability, immunity to electromagnetic interference, high temperature tolerance, and resistance to harsh environments. As a result, different types of fiber optic-based strain sensors have attracted attention from all over the world during recent decades.

Up to now, a great amount of optical fiber strain sensors have been reported and partially commercialized. When concerning the difficulty levels of system structure and measuring principle, the Mach-Zehnder (M-Z) interferometer-based strain sensor is the simplest with very high strain sensitivity, but the cross-effect of temperature and strain is very hard to avoid. To overcome the drawback, a special fiber is used for strain measurement, which has a diameter of only 5 μm. The diameter of this fiber is much smaller than that of a typical single mode fiber (SMF), so that this fiber is extremely insensitive to environmental temperature [6,7]. For realizing the miniaturization of the system structure, the fiber in-line M-Z interferometer has become the main research focus in this field. Currently, the technical methods to realize this kind of fiber in-line M-Z structure are various, like the single-mode-multimode-single-mode structure [8,9], the mode mismatched fusion [10,11], and the waist distorted subuliform fiber [12,13]. Furthermore, a fiber in-line M-Z interferometer is verified to have a strain sensitivity of 0.43 με within the range of 500 με [14]. In addition, a strain sensitivity of 15 με and a tempereture sensitivity of 10 ℃ within the range of 500 με are obtained in another fiber in-line M-Z interferometer [15]. Similarly, the fiber in-line Fabry–Pérot (F-P) interferometer is also a research focus in this field. In terms of fabrication, the structure of the fiber in-line F-P interferometer is easier to realize with natural temperature insensitivity; thus it is more suitable for a fiber-optic strain sensor. At present, the research focus of this technology is to simplify the fabrication process and broaden the shape of the F-P cavity (not limited to a rectangular cavity), in ways such as using micro-electromechanical systems (MEMS) as the two reflecting surfaces of F-P cavity [16,17], using a CO_2_ laser or a femtosecond pulse laser for micro-structure fabrication [18] or the assistant chemical corrosion [19] and so on. Additionally, the fiber in-line F-P interferometer is able to realize a strain sensitivity of 13.5 με [20], a sensitivity of 19.6 με at the range of 600 με  [21], and a sensitivity of 10 με [22]. However, the fiber in-line F-P interferometer is still not suitable for commercial applications right now becasuse of its high cost.

On the contrary, Fiber Bragg Grating (FBG) has become the most mainstream technique, developed in an economic way to be applied in bridges, concrete, and dams for strain measurement. Nevertheless, the cross-effect of temperature and strain is a big issue as well for strain measurement. At present, the commonly used solutions are utilizing speical matrials or structures to make FBG temperature insensitive [23,24] or introducing one more FBG as reference [25]. In addition, the strain sensitivity of FBG could be as high as 3 με. 

Similarly, the Brillouin optical time-domain reflectometer-based (BOTDR) distributed strain sensor is also marred by the cross-effect of temperature and strain [26,27,28], the performance of which has reached a spatial resolution of 5 cm and a strain sensitivity of 63 με [29,30]. At present, the biggest problem with this technology is that the higher the spatial resolution required, the narrower the pulse laser that is needed, which may limit its transmission distance. As a result, the high cost and high requirement of the sensor system make this technology uncompetitive.

Furthermore, with the rapid developement of these sensor technologies, fiber-optic strain sensors have been widely applied for structural monitoring in recent years. Among these applications, BOTDR and FBG techniques are the most commonly used for distributed and quasi-distributed monitoring, respectively [31]. Glisic performed a large scale test on a 13 m long concret pipeline with an exterior diameter of 30.48 cm, and a BOTR-based distributed sensor was placed along its length [32]. The main goal of this work is to achieve real-time monitoring of pipelines that are subject to permanent ground displacements induced by earthquakes. In addition, by addressing the effects of the initial non-circular cross section of a pipeline under internal pressure, the cross deformation of this non-circular pipeline was measured with a BOTDR-based distributed sensor deployed helically on it [33]. Except for pipelines, the health monitoring of bridges is also of great significance. During the construction of the Streicker Bridge, a distributed sensor and many FBG sensors were embedded in the concrete. These sensors work to acquire the important data related to the global performance of the structure, i.e from the early behavior of the concrete to the identification of damage, which is unprecedented [34]. In another application, a multiscale fiber-optic sensing network composed by FBG- and BOTDR-based sensors is employed to realize a large measurement range and to achieve a higher sensitivity [35]. Moreover, in practical applications, the sensing capability could be enhanced by incorporating distribued sensors and FBG sensors, and this combination is effective for achieving a larger measurement range [36].

Compared with all these optical fiber strain sensors mentioned above, the strain sensor proposed in this paper uses a normal single-mode fiber as the sensing and transmission medium, which makes it more cost-efficient and practical. This sensor no longer relies on precise spectrum measurement but realizes strain monitoring by measuring the phase delay induced by strain. Due to the compact size and low price of phase delay detector, this sensor is easy to integrate. In addition, this sensor could achieve a higher strain sensitivity without being affected by the cross-effect of temperature. As a result, when this strain sensor is used for monitoring infrastructures, because of its basic properties, an overall low cost and high precision could be achieved with self-temperature compensation. The working principle of this strain sensor endows itself with a higher environmental tolerance and a low spatial limit. In addition, quasi-distributed strain measurement can be achieved through wavelength division multiplexing and wavelength addressing, so this sensor also shows great potential for long distance structure health and security monitoring.

## 2. Theoretical Basis and Sensing Principle

### 2.1. Theoretical Basis of Fiber-Optic Delay

For a stable optical fiber that is isotropic, uniform, and linear, its optical properties are very stable when not being affected by mechanical stress, and that means that the optical fiber has a constant refractive index profile (RIP). However, when being affected by mechanical stress, the RI of the fiber will be changed, and the variation of RI is the explicit function of the stress tensor. In the Cartesian coordinate system, the strain tensor has six independent components, which are three axial components, $\varepsilon_{xx},\;\varepsilon_{yy},\;\mathrm{and}\;\varepsilon_{zz}$ (*i* = *j*), and three shear components, $\varepsilon_{yz},\;\varepsilon_{zx},\;\mathrm{and}\;\varepsilon_{xy}$ (*i* ≠ *j*). Similarly, the stress tensor also has six independent components, which are $\sigma_{xx},\;\sigma_{yy},\;\mathrm{and}\;\sigma_{zz}$ and $\sigma_{yz},\;\sigma_{zx},\mathrm{and}\;\sigma_{xy}$. Due to Hooke’s law, under the range of elastic deformation, strain is linearly related to stress, and the relationship can be expressed as:

(1) $$\varepsilon_{i}=\sum_{j=1}^{6}h_{ij}\delta_{j}\;(i,j=xx,yy,zz,yz,zx,xy),$$

where each coefficient, according to the symmetry of coordinate axis, has an inner connection as $h_{ij}=h_{ji}$ instead of being absolutely independent. However, in most of the practical instances in which the optical fiber may be affected by mechanical stress, the fiber is commonly placed or embedded in building walls, bridges, concrete, or some other enclosed structure, being used as a sensitive component to realize the measurement of strain or stress [37,38]. Under this condition, because of the special properties and characterizations of these enclosed structures, the shear deformation can be ignored and only the longitudinal stress is concerned ($\sigma_{x}=\sigma_{y}=0$), so that the relationship between strain and stress can be simplified into [39]:

(2) $$\left(\begin{array}{l} \varepsilon_{x}\\ \varepsilon_{y}\\ \varepsilon_{z} \end{array}\right)=\frac{F}{AY}\left(\begin{array}{ccc} 1 & -\upsilon & -\upsilon \\ -\upsilon & 1 & -\upsilon \\ 1 & -\upsilon & -\upsilon \end{array}\right)\left(\begin{array}{c} 0\\ 0\\ 1 \end{array}\right)=\frac{F}{AY}\left(\begin{array}{c} -\upsilon \\ -\upsilon \\ 1 \end{array}\right).$$

Here, *Y* is the Young’s modulus, *υ* is the Poisson’s ratio, *F* is the longitudinal mechanical force, and *A* is the cross-sectional area of fiber. In addition, the variation of RI can be expressed as:

(3) $$\Delta (\frac{1}{n^{2}})=\frac{-2}{n^{3}}\left(\begin{array}{l} \Delta n_{x}\\ \Delta n_{y}\\ \Delta n_{z} \end{array}\right)=\left(\begin{array}{ccc} p_{11} & p_{12} & p_{12}\\ p_{12} & p_{11} & p_{12}\\ p_{12} & p_{12} & p_{11} \end{array}\right)\left(\begin{array}{c} \varepsilon_{x}\\ \varepsilon_{y}\\ \varepsilon_{z} \end{array}\right)=\left(\begin{array}{c} p_{11}\varepsilon_{x}+p_{12}(\varepsilon_{y}+\varepsilon_{z})\\ p_{11}\varepsilon_{y}+p_{12}(\varepsilon_{x}+\varepsilon_{z})\\ p_{11}\varepsilon_{z}+p_{12}(\varepsilon_{x}+\varepsilon_{y}) \end{array}\right).$$

Introducing Equation (2) into Equation (3), the variation of RI could be finally obtained as:

(4) $$\left(\begin{array}{l} \Delta n_{x}\\ \Delta n_{y}\\ \Delta n_{z} \end{array}\right)=\frac{-n^{3}F}{2AY}\left(\begin{array}{c} (1-\upsilon )p_{12}-\upsilon p_{11}\\ (1-\upsilon )p_{12}-\upsilon p_{11}\\ p_{11}-2\upsilon p_{12} \end{array}\right).$$

As for a single-mode optical fiber, only the fundamental mode could transmit in the fiber, and it consists of two modes with perpendicular polarization directions, which are LP^X^_01_ and LP^Y^_01_, respectively. Since the light waves transmitting in the single-mode fiber are substantially transverse waves, the variation of RI approximately satisfies $\Delta n=\Delta n_{x}=\Delta n_{y}$ so as to derive its expression as:

(5) $$\Delta n=\frac{-n^{3}\left[(1-\upsilon )p_{12}-\upsilon p_{11}\right]}{2}\varepsilon_{z}=\gamma n\varepsilon_{z},$$

where *p*_12_ and *p*_11_ are the elastic-optic parameter of fiber and $\gamma =-n^{2}\left[(1-\upsilon )p_{12}-\upsilon p_{11}\right]/2$ can be regarded as the effective elastic-optic coefficient. 

In addition, when transmitting in a certain length of optical fiber, the optical signal will be delayed and the delay is determined by the optical path. If the fiber dispersion is not considered, the delay of the optical signal can be expressed as τ=Ln/c, where *L* is the length of optical fiber, *c* is the speed of light in a vacuum, and *n* is the refraction index of the optical fiber. In fact, fiber optic delay is strain dependent because of its elastic property, so that the variation of delay is:

(6) $$\Delta \tau =\frac{\left(n+\Delta n)(L+\Delta L\right)}{c}-\frac{nL}{c}.$$

Here, Δ*L* and Δ*n* are the change of length and the refractive index of fiber caused by the longitudinal stress, respectively, and the equation meets the condition Δn⋅ΔL<0. Furthermore, because the longitudinal deformation is so small that Δ*L* and Δ*n* are both infinitesimal, the approximate expression ΔL⋅Δn≈0 is obtained. Consequently, the variation of delay can be calculated using Equations (5) and (6):

(7) $$\Delta \tau =\frac{\Delta L\cdot n}{c}(1+\gamma ).$$

In general, the value of the effective elastic-optic coefficient meets, −1<γ<0 and it indicates that the optical fiber delay is supposed to monotonically increase when the fiber is stretched. Here, the elastic coefficient of fiber-optic delay is defined as J=(1+γ)⋅n/c, so Equation (7) could be simplified into Δτ=JLε. Moreover, optical fiber delay is also temperature-dependent due to its thermo-optical property and thermal-expansion property, thus the total differential form of delay is:

(8) $$\Delta \tau =(\frac{L}{c}\frac{dn}{dT}+\frac{n}{c}\frac{dL}{dT})\Delta T,$$

where *T* is temperature. Actually, the first item, dn/dT, stands for the thermo-optical property of the fiber and the second item, dL/LdT, stands for the thermal-expansion property of the fiber. Due to the fact that the value of the thermal-expansion coefficient is much smaller than that of the thermo-optical coefficient for the standard SMF-28 single-mode fiber [40], the thermal coefficient of fiber-optic delay could be similarly defined as K=dn/cdT [41] so as to further simplify Equation (8) into Δτ=KLΔT. Therefore, the variation of fiber-optic delay induced by temperature and strain could be synthetically expressed as:

(9) Δτ=JLε+KLΔT.

### 2.2. Fiber-Optic Delay Based Sensing Principle

**Figure 1:**
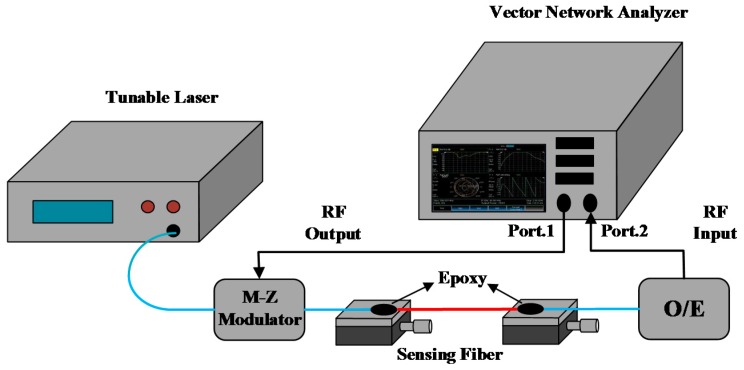
The schematic of the proposed high precision optical fiber delay detection system based on the frequency domain phase method.

According to the theoretical analysis above, a high precision detection system was designed to measure the elastic coefficient and the thermal coefficient of fiber-optic delay, and the schematic of the proposed detection system is shown in Figure 1. The detection system is composed of a vector network analyzer and an optical transceiver module, wherein the vector network analyzer is used to compare the two microwave signals of input and output ports and then the inner calculating module of vector network analyzer can work on translating the phase difference between the two microwave signals into the time delay of the entire measuring transmission optical path. 

More specifically, the offset compensation function of the vector network analyzer is utilized during the practical measurement process, and the basic operation could be described as: the optical signal is modulated by the microwave signal from port.1; after that, the modulated optical signal transmits through the measuring optical path and is de-modulated into a microwave signal, importing into port.2. By sampling and calculating, the phase difference between the input and output microwave signals could be obtained, and the vector network analyzer could work on introducing extra time delay to compensate the measured phase difference. Therefore, the phase difference between the two signals can be finally eliminated by turning the compensation time delay, and the current compensation value is equal to the time delay of the measuring optical path.

**Figure 2:**
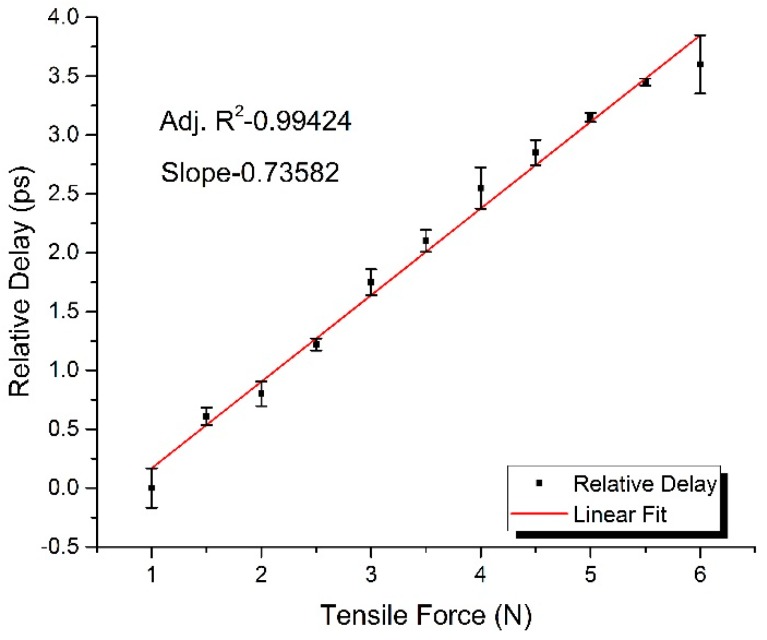
The experimental response of relative delay for the rise of the longitudinal tensile force.

By using the method above, the relative delay of fiber was measured by the vector network analyzer within a tensile force range of 1 N to 6.5 N and with an increment step of 0.5 N. Figure 2 demonstrates the experimental response of the relative delay for the rise of the tensile force, and the variation was recorded with an increment of 0.5 N. Furthermore, the linear fit of the elastic property of fiber-optic delay is presented in Figure 2 as well, wherein the standard SMF-28 type single-mode fiber, produced by Corning Inc. (New York, NY, USA), was chosen as the measuring fiber for which the refractive index is is about 1.468, Young’s modulus is 65 GPa, the core diameter is 125 μm, and the initial unstressed length is 15.5 cm. The experimental results indicate that the measured elastic coefficient is 0.736 ps/N and the relative delay linearly increases with the increment of longitudinal strain, which is a proof of the theoretical analysis above. According to Hooke’s law, the longitudinal strain is ε=F/(Y⋅A), so the elastic coefficient could be further obtained as *J* = 3.78 ps/km·με. As for the material of the fiber, the elastic parameters of which are *p*_11_ = 0.113, *p*_12_ = 0.252 and *υ* = 0.17, its effective elastic-optic coefficient is *γ* = −0.22. Combined with J=(1+γ)⋅n/c, the elastic coefficient is calculated as *J* = 3.82 ps/km·με with an experimental error of 0.04 ps/km·με.

**Figure 3:**
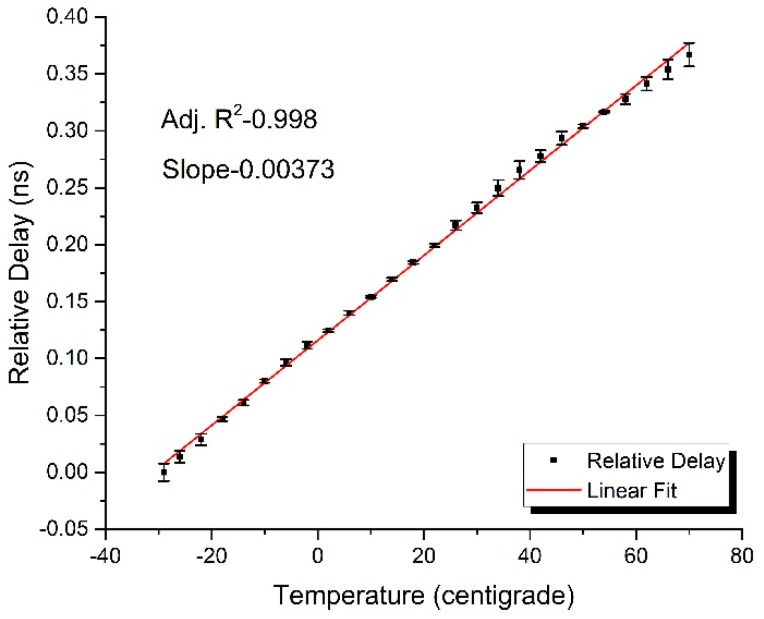
The experimental response of relative delay for the rise of temperature.

Based on the same experimental setup presented in Figure 1, the thermal coefficient of fiber-optic delay was measured in the temperature range from −30 ℃ to 70 ℃ with an increment step of 3 ℃ by utilizing the same optical fiber as in the measuring sample, the initial length of which is 95.15 m. Figure 3 presents the experimental response of delay difference for the rise of temperature and indicates a thermal coefficient of about *K* = 39.2 ps/km·℃.

As a result, on the basis of Equation (9), if the initial unstressed length *L* and elastic coefficient *J* of a sensing fiber are known, the strain can be measured by monitoring the change of fiber-optic delay in an experimental environment with a stable temperature.

## 3. Design and Analysis of Strain Sensor

### 3.1. Design of Strain Sensor

Based on the discussion above, the relationship between delay and strain is set up, and the strain could be measured by detecting the change of delay. However, due to Equation (9), fiber-optic delay is both strain-dependent and temperature-dependent, so the measurement of strain will be affected by the cross-effect of temperature as well. To solve this problem, an extra reference optical path is introduced to predigest this process by detecting the delay difference of two optical signals instead. This schematic process can be presented as:

(10) $$\tau_{1}-\tau_{0}=JL\left(\varepsilon_{1}-\varepsilon_{0}\right)\to \left(\tau_{1}-\tau_{2}\right)-\left(\tau_{0}-\tau_{2}\right)=JL\left(\varepsilon_{1}-\varepsilon_{0}\right).$$

Here, *τ*_0_ and *τ*_1_ are the delays of the sensing fiber at strain *ε*_0_ and *ε*_1_, respectively, and *τ*_2_ is the delay of the reference fiber. Furthermore, the delay of the optical carrier wave is linearly related to the phase of the modulating signal with a proportionality coefficient of 2π*f*, so that Equation (10) could be further transformed into:

(11) $$\Delta \phi_{1}-\Delta \phi_{0}=2\pi fJL\left(\varepsilon_{1}-\varepsilon_{0}\right),$$

where Δ*φ*_0_ and Δ*φ*_1_ are the obtained phase differences at strain *ε*_0_ and *ε*_1_, respectively, and *f* is the frequency of the modulating signal. Consequently, the measurement of strain could be finally achieved by the detection of phase difference so as to simplify the measurement process.

In practical terms, the environmental temperature is quite hard to keep stable, and the variation of temperature is an insurmountable cross-effect for the measurement of strain. Thus Equation (11) could be further rewritten as:

(12) $$\Delta \psi =2\pi fJL\left(\varepsilon_{1}-\varepsilon_{0}\right)+2\pi fK\Delta L\cdot \Delta T(t),$$

where Δ*ψ* is the variation of phase difference, Δ*L* is the length difference between sensing path and reference path, and Δ*T*(*t*) is the temperature function related to time. Hence, another advantage of the introduced reference path is to compensate for the effect of temperature on the sensing path, which is beneficial for this strain sensor to resist the environmental temperature fluctuations. In addition, the lengths of sensing path and reference path are urged to be equal.

**Figure 4:**
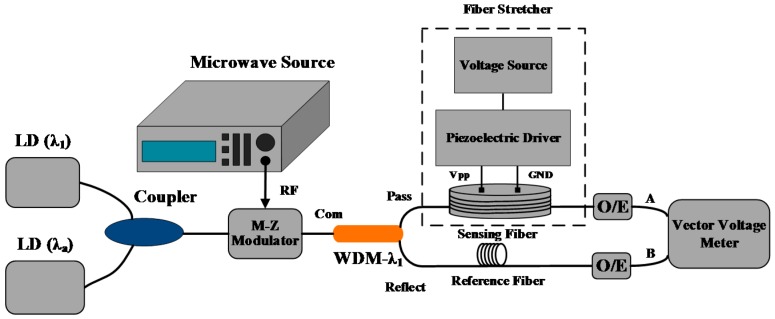
The schematic structure of a fiber-optic delay-based strain sensor.

According to the principle above, a fiber-optic delay based strain sensor is depicted in Figure 4. In this sensor, the optical transmitter is composed of two distributed feedback (DFB) lasers with fixed wavelengths of *λ*_a_ and *λ*_1_, respectively. The two light beams are coupled and modulated and then pass through the sensing areas behind after being divided by a wavelength division multiplexer (WDM). The WDM used in this sensor is a three-port device, including a com port, a pass port and a reflect port. The com port is accessible for all wavelengths and is connected to a transmission optical fiber. The pass port is similar to a filter for the wavelength of λ_1_, being connected to a sensing fiber, so the light beam λ_1_ is regarded as the sensing light. On the contrary, the reflect port is accessible to all wavelengths except for *λ*_i_; thus it is connected to the reference fiber, and the light beam *λ*_a_ is used as the reference light. After that the two light beams are de-modulated and imported into a Vector Voltage Meter (VVM), which is used to measure the phase difference between the two input microwave signals with high precision. In addition, a piezoelectric ceramics-based optical fiber stretcher, with a controlling voltage range of 0–120 V and a radial deformation resolution of 4 nm, was used to realize the generation and control of strain in this sensor.

**Figure 5:**
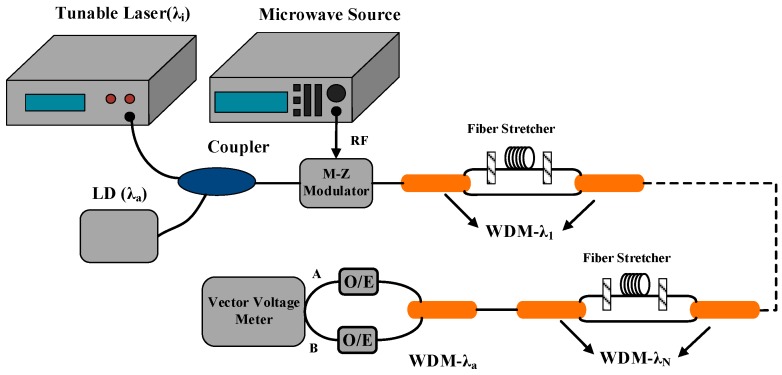
The fiber-optic delay based quasi-distributed strain sensor.

One significant advantage of the designed sensor is that it is cascade-able, which means that a quasi-distributed strain sensor could be simply realized by connecting several basic sensor units in series with slightly modifications. Figure 5 presents the schematic structure of the quasi-distributed strain sensor, in which a DFB laser is replaced by a tunable laser whose wavelengths is *λ*_i_ (i = 1–N) and there are a pair of WDMs being placed beside each sensing area. In addition, by changing the output wavelength of the tunable laser, each sensing area could be measured in sequence.

### 3.2. Calibration of Strain

**Figure 6:**
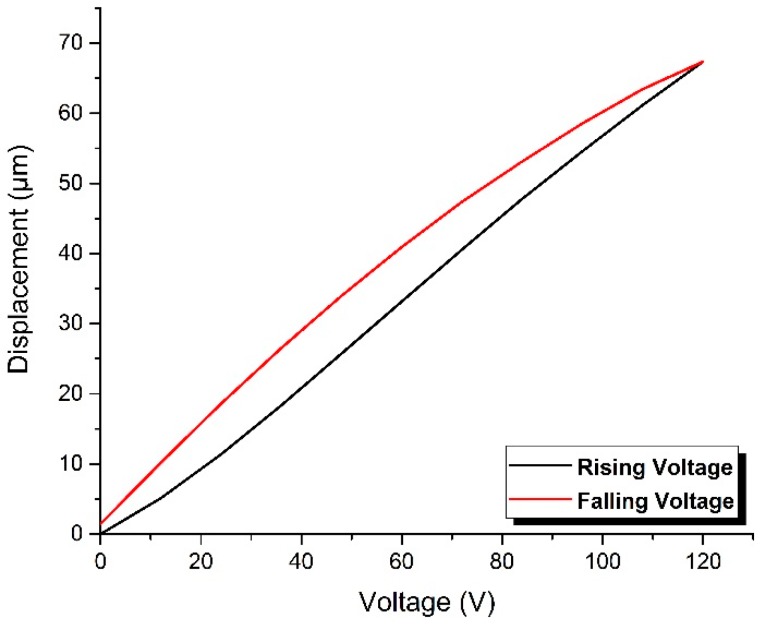
The testing results of radial displacement of this fiber-optic stretcher under the controlling voltage range of 0–120 V.

Before the sensing experiment, the radial displacement of the stretcher in the controlling voltage range of 0 V–120 V was tested, and the obtained data is presented in Figure 6. The experimental results demonstrate that (1) its radial displacement is not linear with controlling voltage and (2) during the rising and falling processes of controlling voltage, the radial displacement at each testing voltage point is different, and the displacement curve during the falling process of voltage has a bigger curvature. Therefore, when the fiber-optic stretcher is used for strain control, every testing point must be calibrated in advance, and the falling process of voltage is more suitable to be chosen for directly verifying the accuracy of the calibration result. 

Moreover, for the reason that the sensing fiber coiled on the optical fiber stretcher may impede its radial displacement, the practical stretched length of the sensing fiber will deviate from the theoretical value, so that we calibrated at each testing point in the voltage range of 102−24 V with a decrement step of 6 V, using the experimental setup presented in Figure 1. The number cycles of the sensing fiber coiled on this stretcher is 14, which means that the initial unstressed length of sensing fiber is 3.08 m. As the variation of time delay induced by the change of controlling voltage is specially required, the measured time delay at 24 V was set as a zero reference, replacing the absolute value with a relative value so as to simplify the subsequent data processing.

**Figure 7:**
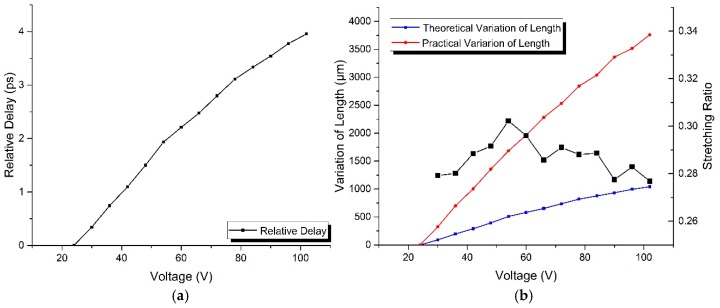
The calibration of strain in the voltage range of 102–24 V with a decrement step of 6 V: (**a**) The response of relative delay to voltage; (**b**) The response of variation of length to voltage.

Figure 7a demonstrates the experimental response of relative delay for the decrease of controlling voltage. If the frequency of the modulating signal is 5 GHz, the theoretical measurement precision of the strain-induced fiber-optic delay is about 100 fs for this sensor, whereas the measurement precision of the vector network analyzer is as high as 1 fs. As a result, the system error induced by the calibration is less than 1%.

As for the sensing fiber coiled on the piezoelectric ceramics-based optical fiber stretcher, the following relationships could be set up as [42]:

(13) $$\begin{array}{c} \Delta \tau =JL\varepsilon =J\Delta L_{P}\\ \Delta L_{T}=2\pi \cdot Q\cdot (\Delta r_{i}-\Delta r_{24})(i=0-120)\\ R=\Delta L_{P}/\Delta L_{T} \end{array}.$$

**Figure 8:**
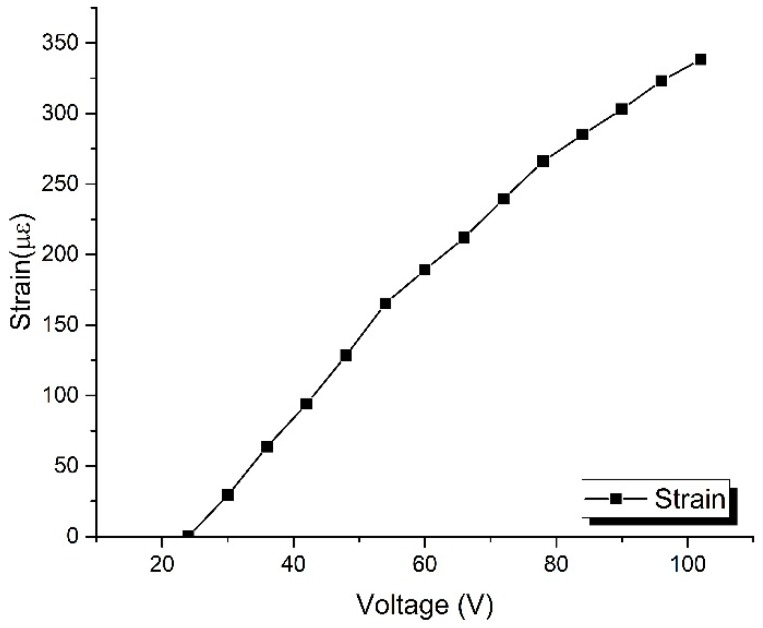
The response of strain to the decrease in controlling voltage.

Here, Δ*L*_P_ and Δ*L*_T_ are the practical and theoretical stretched lengths of the sensing fiber, respectively, *J* is the elastic coefficient of the fiber-optic delay whose value is about *J* = 3.8 ps/km·με, *Q* is the cycles of the coiled sensing fiber, Δ*r*_i_ is the radial displacement of the stretcher at the controlling voltage *i*, and *R* is defined as the stretching ratio. The obtained data is presented in Figure 7b, and the results indicate that the practical stretched length of the sensing fiber is less than its theoretical value with a stretching ratio of about 0.29 at each testing voltage point. In addition, the measured value of the stretching ratio is approximately normally distributed, and its peak point corresponds to the position with the biggest curvature of the displacement curve in Figure 8, which could be regarded as a verification of the accuracy of the calibration. Furthermore, the response of strain to the decrease in the controlling voltage is finally obtained and presented in Figure 8, and the whole experimental process was conducted in a room with a constant temperature.

## 4. Experimental Results and Discussion

**Figure 9:**
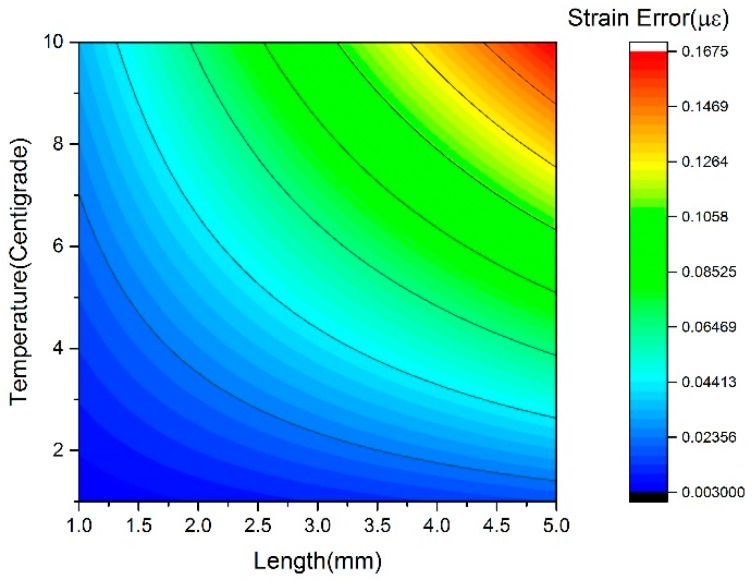
The strain error induced by temperature fluctuations.

A prototype experimental setup for testing the feasibility and practicability of the strain sensor presented in Figure 5 was built. In this sensor, the output wavelengths of the tunable laser and the DFB laser are *λ*_i_ = *λ*_1_ = 1549.32 nm and *λ*_a_ = 1552.12 nm, respectively. The frequency of the modulating signal is 5 GHz, and the bandwidth of the M-Z modulator and the two photodetectors is 10 GHz. The vector voltage meter works to measure the phase difference between the two microwave signals of port A and port B with a measurement precision of 0.1°, which is equivalent to a strain resolution of about 4.75 με. Due to the concerning temperature fluctuations, the impact of temperature on the measurement of strain must be evaluated as well. According to the initial parameters, the elastic coefficient is *J* = 3.8 ps/km·με, the thermal coefficient is *K* = 39.2 ps/km·℃, and the unstressed length of sensing fiber is 3.08 m. Combined with Equations (9) and (12), the strain error induced by temperature fluctuations is discussed under the conditions of Δ*L* = 1 mm − 5 mm and Δ*T* = ± 10 ℃, and the simulation results are presented in Figure 9. Thus, to achieve a strain error of less than 1% (0.0475 με) under the maximum temperature fluctuation of 10 ℃, the length difference between sensing path and the reference path should be controlled by less than 1.4 mm.

Currently, the cutting precision of fiber can be controlled under one millimeter in our lab. In this experiment, the delay of the sensing fiber is 35.778 ns, and that of the reference fiber is 35.781 ns, which is equivalent to a length difference of about 0.6 mm. Thus, the length difference between the two paths is low enough to meet the requirement of the sensor.

**Figure 10:**
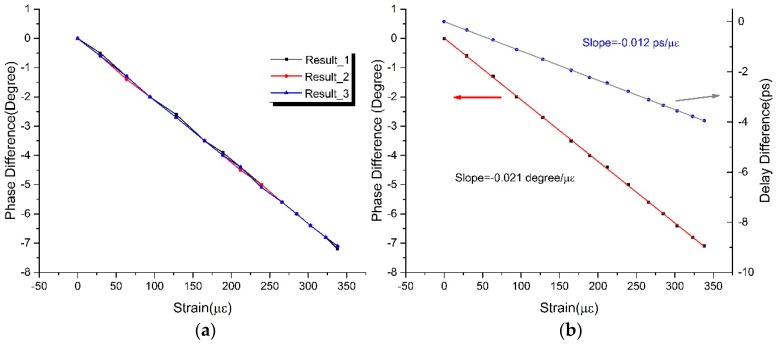
The experiments for testing the strain sensitivity of this sensor: (**a**) The repeatability response of phase difference under the same experimental conditions; (**b**) The response of degree and delay of this sensor with respect to a change in strain.

Depending on the analysis above, the phase difference of port B and port A (A/B) was measured in the voltage range of 102−24 V with a decrement step of 6 V, and the measured value at voltage 24 V was set as the zero reference. Meanwhile, to test the repeatability of this sensor, the experimental process was repeated many times with the same voltage range and decrement in a room with a constant temperature. Combined with the calibration in Section 3, the repetition results of this experiment are presented in Figure 10a, which show that a similar variation of phase difference is found with respect to a change in strain. 

The response of degree and delay of this sensor with respect to a change in strain was finally obtained by multiple measurements and equalization, as shown in Figure 10b. The experimental results demonstrate that phase difference is linear with strain, which conforms to the previous theoretical results. Moreover, the linear fitting coefficients of phase and delay are −0.021 °/με and −0.012 ps/με, respectively. As a result, the sensor with the measurement precision of 0.1° has a strain sensitivity of 4.75 με in the range of 350 με.

**Figure 11:**
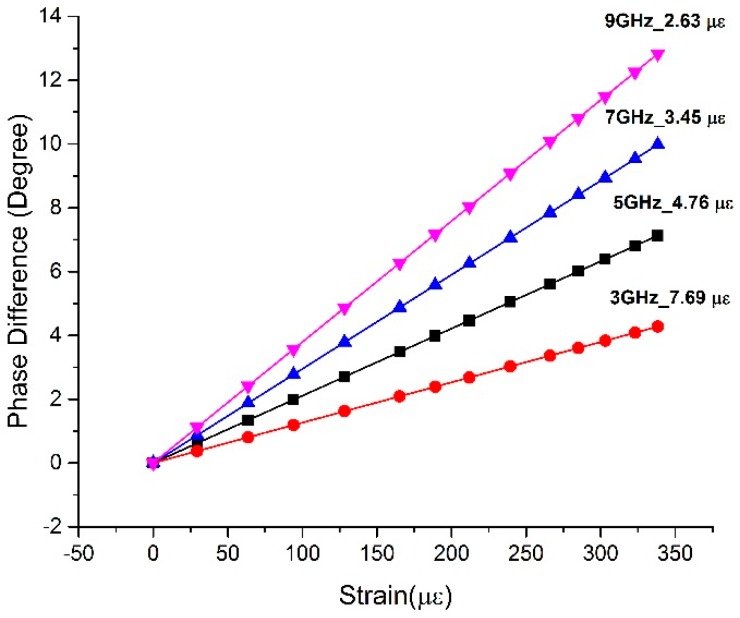
The strain sensitivity of a sensor working at different frequencies.

As for a known strain sensor, the length of the sensing fiber and the measurement precision of the detector are specific; thus the effective way to improve its strain sensitivity is by raising the working frequency of the modulating signal. Figure 11 simulates the response of the phase at different working frequencies, which shows that the increase of working frequency is truly effective in improving the strain sensitivity of the sensor. In addition, during the period of design, a longer sensing fiber and higher measurement precision also work to make the sensor more sensitive to strain.

**Figure 12:**
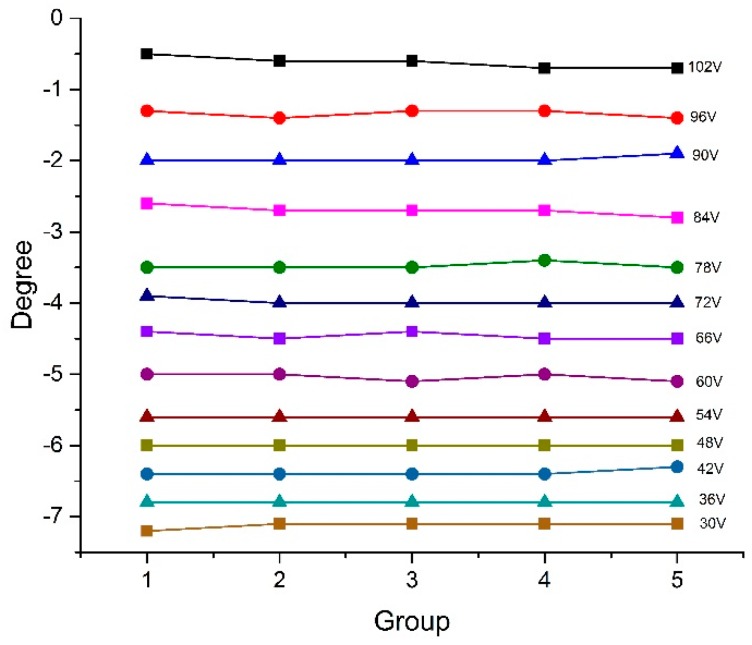
The temperature insensitive response of phase difference at each controlling voltage.

To verify its temperature insensitivity, this sensor was placed in an open environment, which caused the sensor to be continuously affected by the unstable and random environmental temperature fluctuations. Under this condition, each controlling voltage was maintained for more than 20 min and measurement was repeatedly conducted several times during this period, and the obtained response is presented in Figure 12. The experimental results show an average degree fluctuation of less than 0.025° at each controlling voltage. On basis of the discussion above, this degree fluctuation obviously does not result from the unstable temperature and is more likely caused by the unstable working performance of the fiber stretcher and could be eliminated by averaging the multiple measured values. In this case, temperature fluctuations will no longer be a problem in practical applications.

## 5. Conclusions

In this paper, a high precision temperature insensitive strain sensor based on optical fiber delay was reported, which is able to realize the measurement of strain by detecting delay instead of spectrum because of the elastic property of fiber-optic delay. A prototype experiment to verify its feasibility was conducted and the experimental results prove it to be feasible and practical with high precision and temperature insensitivity. To achieve an overall low cost, the vector voltage meter utilized in this paper could be simply replaced by a phase discriminator, which is increasingly mature and can be simply implemented based on functional chips with high precision. In addition, because of its working principle, the sensor has a great potential to be a substitution in the field of long distance structure monitoring. Further experiments on this study will be conducted.
